# The Reliability of Parkour Skills Assessment

**DOI:** 10.3390/sports6010006

**Published:** 2018-01-24

**Authors:** Martin Dvořák, Jiří Baláš, Andrew J. Martin

**Affiliations:** 1 Faculty of Physical Education and Sport, Charles University, Prague 16252, Czech Republic; balas@ftvs.cuni.cz; 2 School of Sport, Exercise & Nutrition, Massey University, Palmerston North 4442, New Zealand; a.j.martin@massey.ac.nz

**Keywords:** obstacle course, objectivity, Krippendorff’s α, Mann-Whitney test

## Abstract

The aim of the study was to determine the reliability of parkour skills assessment in field conditions. Twenty young men completed three trials of a parkour obstacle course on two separate days. The tested group consisted of 10 beginners (age 16 ± 1 years, body mass = 65 ± 12 kg, height = 177 ± 7 cm) and 10 advanced traceurs (age 18 ± 2 years, body mass = 68 ± 14 kg, height = 178 ± 6 cm). The performance was video-recorded and subsequently analyzed by three raters (total score 0–45). Median and percentiles were used to characterize results from all sessions by all raters. Inter-rater, intra-session and inter-session reliability were assessed using Krippendorff’s α for ordinal data. The Mann-Whitney test was used to assess the differences between beginners and advanced traceurs. Advanced traceurs obtained a total score from 41 to 44 whilst beginners achieved 27 to 33 points. Krippendorff’s α for total score ranged from 0.910 to 0.916 between raters, 0.828 to 0.874 between trials, and from 0.839 to 0.924 between days. The proposed parkour course differentiated two different ability levels and the skills assessment demonstrated excellent reliability between raters, trials, and days.

## 1. Introduction

Parkour has been growing in popularity in recent years, and is practiced by many young people in the world [1]. A traceur (or parkourist = parkour practitioner) has to pass over obstacles in an urban/natural environment or in a special parkour facility by means of his own body abilities. Parkour basically involves running in combination with specific techniques of jumping, climbing and quadrupedal movements [2,3,4,5,6]. Contrary to the image depicted by some popular videos on the internet, parkour can be considered as a safe activity when practiced under expert supervision [7]. Moreover, parkour is recommended as a suitable activity in secondary and high school education [8,9,10]. It has been shown that parkour has a beneficial effect on physical fitness [3,11,12,13], mental health and social learning [14,15,16,17,18,19].

However, changes in motor skill development have not yet been documented, although they are a fundamental part of parkour training. Motor skill development is often neglected in the training process and in physical education, although it has a positive direct relationship to various health outcomes [20]. School aged children who are able to perform fundamental motor skills such as running, jumping, throwing, kicking, or catching at high levels of proficiency are more physically fit, less obese, and less at risk of hypokinetic disease [21]. It has been suggested that the development of motor skill competence in a variety of forms across childhood may provide a foundation for promoting positive trajectories of fitness and physical activity behaviour across childhood, adolescence and into adulthood [22]. Moreover, children from the beginning of their school education, as well as adolescents who have highly developed motor skills, are able to more actively participate in sports and physical education lessons [23,24]. Until now, there is a lack of evidence on parkour skill development in children despite their beneficial effect in everyday life [25,26]. 

The importance of motor skill competence is well documented in the existing literature; however, assessing motor skills poses several methodological problems [27,28]. One of the critical points in the physical education process is to assess “how” children perform motor skills [29,30,31,32]. Numerous authors have addressed the important role of qualitative assessment when learning new motor skills [31,33,34,35,36,37,38].

As parkour has been expanding in school and sport education; it has become interesting for sport teachers, personal coaches and group trainers [3]. Including a measurement tool in parkour training might help to indicate the level of motor development or degradation [39]. To assess the level of parkour skills in practice, standardized evaluation needs to be designed. The aim of this study was to propose a reliable assessment of parkour skills in field conditions.

## 2. Materials and Methods

### 2.1. Subjects

Twenty young men volunteered for the study. Ten participants with no previous parkour experience were considered as beginners ((mean ± SD) age 16 ± 1 years, body mass = 65 ± 12 kg, height = 177 ± 7 cm), and ten participants with at least 6 months (range: 6 months–2 years) of parkour history were considered as advanced traceurs (age 18 ± 2 years, body mass = 68 ± 14 kg, height = 178 ± 6 cm). The experimental procedures were communicated to all study participants and their parents if under-age, who then signed informed written consent to allow participation in the study. The Ethics Committee of the Faculty of Physical Education and Sports, Charles University in Prague has approved the experiment in accordance to the Declaration of Helsinki.

### 2.2. Procedure

**Figure 1:**
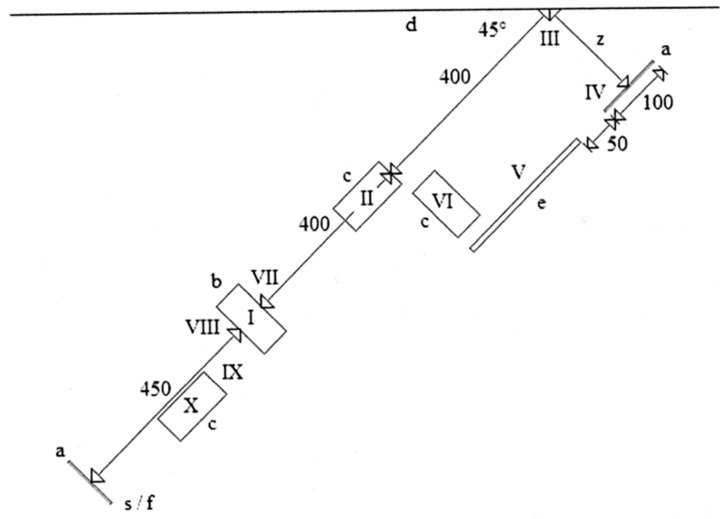
Parkour obstacle course layout (dimensions are given in centimetres). Adhesive tape 5 cm wide (a), wooden vaulting box (b), mattresses (c), wall (d), wooden bench (e), precision jump distance (z). Consecutive sequence of parkour techniques: start/finish line (s/f), monkey vault (I), parkour roll (II), tic-tac (III), precision jump (IV), cat balance (V), parkour roll (VI), step vault (VII), corkscrew pop-up (VIII), landing (IX), parkour roll (X).

On the first day, participants came to the gym and performed 3 trials of a parkour obstacle course. After 2 days, participants completed another 3 trials of the same parkour obstacle course. At the beginning of each visit, participants warmed up and familiarized themselves with the obstacle course and performed 3 practice trials. The parkour course shown in Figure 1 consisted of commonly used parkour techniques—monkey vault, parkour roll, tic-tac, precision jump, cat balance, step vault, corkscrew pop-up, parkour landing. Each technique was described in detail by Edwardes [40]. The participant started from a standing position by verbal command from a tester. The run-up was followed by a monkey vault performed over the vaulting box. Right after the landing, a parkour roll was performed onto a mattress. A run-up followed and the wall was hit to execute a tic-tac. The distance between the wall and the landing area was overcome in the air. A precision jump was used to land precisely on a marked point. A quadrupedal movement was then used to go over a bench. A parkour roll was initiated from the squat position on the bench and performed on the mattress. A run-up followed and a step vault was performed over the vaulting box. Right after the landing, a 180° turn was done and a vaulting box was mounted using the technique of corkscrew pop-up. The landing was performed followed by a parkour roll on the mattress. The run to the finish line was the last task. The end of performance was considered at the same time when the participant crossed the finish line by the trunk. The time was measured to support the motivation of participants. All trials were video-recorded to allow later assessment.

The parkour obstacle course was assembled in a gym, where a wooden vaulting box (with adjustable height), a wooden bench (the narrow part was used for cat balance), and three mattresses were the only equipment used. The vaulting box and the bench were fixed to the floor by adhesive elements to prevent sliding. The obstacle course was set as shown in Figure 1. The settings of this course aimed to facilitate easy reproducibility. Three measures related to body dimensions and actual physical fitness level were considered while assembling the obstacle course:

1. The height of the vaulting box used for the techniques of monkey vault, step vault and corkscrew pop-up was set to 100 cm. It represented, approximately, the mean hip height of participants.
2. The take-off area marked on the wall where the tic-tac technique was initiated was defined between 100 and 150 cm (50 cm wide). It represented the distance between the mean hip height and the mean shoulder height of participants. The area should have a non-slip surface.
3. The distance between the wall where the take-off area for the tic-tac and the landing line was marked on the floor. Where the precision jump was terminated was set to 200 cm. It represented 4/5 of the mean value for the current standing long jump performance of participants. The distance was measured from the point right below the centre of the take-off area close to the floor to the centre of the landing line for the precision jump.

### 2.3. Data Collection

Participants performed the parkour obstacle course under the administration of two testers. One tester instructed participants and measured the time, and the other one video-recorded the performance. The camera (Canon Inc., Ōta, Japan; 60 frames per second) was placed with a side view to best detect the particular skill performance whenever circumstances permitted. The skills were administered in the order of the scoring sheet, as depicted in Table 1.

### 2.4. Data Analysis

Three raters with practical parkour training experience evaluated independently the parkour skills from the video record according to evaluation criteria (Table 1). Ten consecutive parkour techniques and 30 specific movements in particular sequence were assessed. When the parkour technique was performed, 1 point was recorded and the specific movements belonging to this technique were further evaluated as follows. When the specific movement was performed correctly, 1 point was recorded, when the specific movement was not performed correctly, 0 points were recorded. When the parkour technique was not performed at all then 0 points were recorded. The specific movements belonging to this technique were not evaluated and the following parkour technique was evaluated. In total, 45 points could be obtained (scale from 0 to 45): 0 to 10 points for performance of parkour techniques, 0 to 30 points for performance of specific movements and 0 to 5 points for the fluency of the participants’ overall movement manifestation. The normality was assessed by the Kolmogorov-Smirnov test and all ratings showed normal distribution. However, due to the dichotomous and ordinal nature of the scale, non-parametric statistics were performed. Median and percentiles were used to characterize data from all sessions in all raters. Inter-rater, intra-session and inter-session reliability were assessed using Krippendorff’s α. Krippendorff’s α was used to assess reliability as it is suitable for dichotomous and ordinal data regardless of the number of observers, levels of measurement, sample sizes, and presence or absence of missing data. Krippendorff’s α can fluctuate between 0 and 1.0, where 0 shows no reliability, and 1.0 shows perfect reliability [41]. Hayes and Krippendorff [41] proposed values above 0.8 to indicate good reliability, 0.67–0.8 low reliability, and <0.67 really low reliability. The Mann-Whitney test was used to assess the differences between groups. Statistical significance was set to *p* < 0.05. Statistical computations were performed using IBM SPSS for Windows (version 22, IBM, Chicago, IL, USA).

## 3. Results

**Figure 2:**
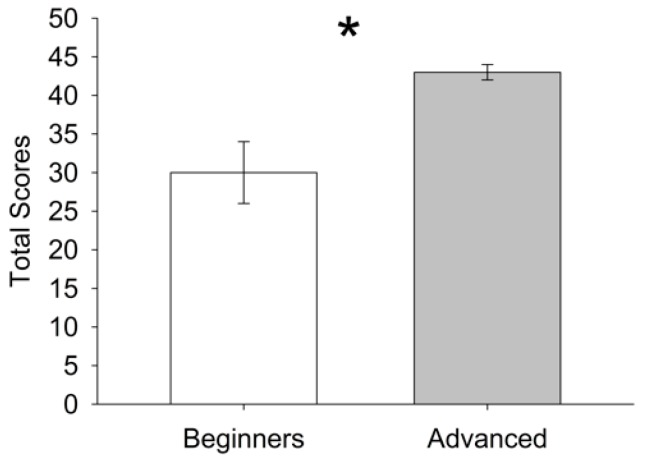
The difference in parkour skills between beginners and advanced traceurs shown as a median (25th/75th percentile). * = significantly different.

The differences in total scores between advanced traceurs and beginners are shown in Table 2. The total scores for advanced traceurs ranged between 42 and 44 and were significantly higher (*p* < 0.001) than the total scores for beginners ranging from 27 to 34 (Figure 2). Krippendorff’s α for total score between raters ranged from 0.910 to 0.916, for total score between trials from 0.828 to 0.874, and for total score between days ranged from 0.839 to 0.924 (Table 3).

## 4. Discussion

The main aim of this study was to determine the reliability of parkour skills assessment in field conditions. To our knowledge, this is the first study to propose inter-rater, intra-session and inter-session reliability of parkour skill assessment. The main finding of the study is that the proposed parkour obstacle course differentiated ability levels and the obtained total scores provided excellent objectivity and test-retest reliability. The known group difference method could be used to establish construct validity in this study [42]. However, to establish the validity of the parkour obstacle course was not the main aim of this study. 

Krippendorff’s α was used to assess reliability as it is suitable for dichotomous and ordinal data regardless of the number of observers, levels of measurement, sample sizes, and presence or absence of missing data [41]. Krippendorff’s α can fluctuate between 0 and 1.0, where 0 shows no reliability, and 1.0 shows perfect reliability [41]. The authors proposed values above 0.8 to indicate good reliability, 0.67–0.8 low reliability, and <0.67 really low reliability. The Krippendorff’s α values related to raters were 0.927 from both sessions and indicated good congruent results among the three raters in our study. Agreement among raters who use the same instrument needs to be positive and high to assure reliability of the results [43]. Higher values in outcomes for raters in our study may be partly explained by the use of video recording. Video recording allows more detailed scrutiny and flexibility when carrying out assessments. Videos may be played several times if needed, and slow-speed replay can assist the observation of performance criteria that are difficult to observe without slow motion [44]. Despite different statistical methods used Barnet and colleagues [45] reported intra–class correlation (ICC) = 0.93, based on live observation for inter-rater reliability in test of object-control skills. Whilst the study of Slotte [46] analyzed the same test through video recordings and reported slightly higher value of ICC = 0.995.

With regards to reliability between trials, Krippendorff’s α was 0.853 in our study. It can still be considered as a good reliability [41]. Regarding between days reliability, Krippendorff’s α values ranged between 0.839 and 0.924 in the current study. In comparison to Barnett and Sam [47] who found ICC = 0.60 for test-retest in golf skills, our study seems to provide more reliable results despite the different statistics used. We found similar results to Faber [48] who reported ICC = 0.80 for agility table tennis subtest or Rubajczyk [49] who reported ICC between 0.759 and 0.908 for soccer game-related skills in 12 to 15 years old boys. 

It has to be noted that high reliability in our study might have been improved by low discriminative ability of the scale in advanced traceurs who achieved top scores, and therefore, the variability of their scores was low.

The main limitation of the current study is a relatively small and homogenous sample size in the two groups. The current results were limited to males, age 15–20 years, therefore the current results may not be representative for females or the other age groups. Further studies need to examine these populations. Moreover, the distinguishing capacity of the obstacle course and evaluation sheet might not be sufficient in highly advanced traceurs, as the obstacle course consisted of basic skills. The parkour course and evaluation sheet were proposed to be used rather in parkour beginners than in advanced traceurs. The high scores in advanced traceurs have very low discriminative ability. Therefore, a modification of the obstacle course for this population should be applied. The possible modification of the evaluation criteria for advanced traceurs is to add the time of performance or modulate the distance of the jumps or height of the obstacles. The obstacle height of the vaulting box may affect the performance of the monkey vault, step vault and corkscrew pop-up in persons with lower body height, and individual obstacle height adjustment is necessary for a heterogeneous sample of participants. Despite several limitations of the study and qualitative assessment of parkour skills, the strengths of this study should also be mentioned. Three raters assessed six trials in two days and several reliability measures are provided. The parkour course and evaluation procedure are easy to replicate and can be set in field conditions. Therefore, the proposed parkour course and evaluation procedure seems useful to assess parkour skill level in school or in extracurricular education.

## 5. Conclusions

The proposed parkour obstacle course with an evaluation sheet were shown to be reliable tools for parkour skill assessment in field conditions. The presented results demonstrated excellent reliability between raters, trials as well as between days and, therefore, the parkour course with the evaluation procedure seem suitable for parkour skills assessment in 15–20-year-old boys.

## Figures and Tables

**Table 1:** Parkour obstacle course evaluation criteria.

Parkour Technique	Performed = 1 point/Not performed = 0 points
Specific Movement	
**Monkey vault—overcome the vaulting box**	
take-off from both feet or split-foot take-off	
both palms squarely placed to the top of the box	
legs are tucked tight against the body with no contact with the box	
land on the balls of feet	
knee flexion is 90° and more	
**Parkour roll—perform the roll**	
roll right after the landing, body lean forward, hands placed to the floor	
roll over the front shoulder and down to the opposite hip (not over the head)	
**Tic-tac—push away from the wall**	
take-off from the leg further from the wall	
the leg is placed to the wall to the area between the hip and the shoulder height	
after push off from the wall head and body is turned actively to the landing line	
**Precision jump—overcome the distance between the wall and the line by air**	
reach forward with both feet while passing the middle of the jump	
land on the balls of feet	
knee flexion is 90° and more	
balls of feet are precisely placed to the landing line, final position is balanced	
**Cat balance—overcome the obstacle with no touch with the floor and touch the end of the bench by hand**	
knees are not higher than ankles	
simultaneous movement of the left upper limb and the right lower limb (opposite the same)	
**Parkour roll—perform the roll**	
roll right after the landing, body lean forward, hands placed to the floor	
roll over the front shoulder and down to the opposite hip (not over the head)	
**Step vault—overcome the vaulting box**	
take-off from one foot	
place the same hand to the box while bringing the other leg to step up onto the box	
trailing leg comes up and through the space between the body and the box with no contact with the box	
trailing leg touches the ground first	
**Corkscrew pop-up—get on top of the box**	
place both hands on the box, one is inverted so that the fingers point back towards the body	
jump up and spin 180° around the arm with inverted palm	
legs are not in contact with the box while rotating the body	
final squat position on the balls of feet near the front side of the top of the box	
**Parkour landing—drop from the box**	
land on the balls of feet	
knee flexion is 90° and more	
**Parkour roll—perform the roll**	
roll right after the landing, body lean forward, hands placed to the floor	
roll over the front shoulder and down to the opposite hip (not over the head)	

**Table 2:** Total scores for parkour obstacle course shown as a median (25th/75th percentile). Raters (R1, R2, R3), trials (T1, T2, T3), advanced traceurs (A), beginners (B).

-	T1A	T2A	T3A	T1B	T2B	T3B
**Session 1**						
R1	43(42/43)	43(41/43)	44(43/44)	33(27/35)	29(26/32)	29(22/32)
R2	43(43/43)	42(40/44)	44(43/44)	34(28/35)	28(26/31)	27(22/31)
R3	42(40/43)	43(40/43)	43(43/44)	34(29/38)	31(29/35)	32(23/34)
**Session 2**						
R1	44(43/45)	44(43/44)	44(42/44)	32(24/34)	29(25/34)	31(27/33)
R2	44(44/44)	44(42/45)	44(43/44)	29(24/32)	29(24/32)	31(25/34)
R3	44(42/45)	44(43/44)	44(42/44)	32(26/35)	32(26/33)	33(28/36)

**Table 3:** Krippendorff’s α for reliability measures and raters (R1, R2, R3).

-	α	95% α
Inter-rater		
Session 1	0.910	0.878–0.937
Session 2	0.916	0.865–0.955
Both sessions	0.927	0.882–0.963
Intra-session		
R1	0.840	0.784–0.891
R2	0.874	0.822–0.916
R3	0.828	0.768–0.882
All raters	0.853	0.802–0.898
Inter-session		
R1	0.839	0.732–0.930
R2	0.924	0.874–0.967
R3	0.844	0.744–0.929
All raters	0.846	0.745–0.934
